# Breast Cancer Incidence Patterns in the Saudi Female Population: A 17-Year Retrospective Analysis

**DOI:** 10.3390/medicina58111617

**Published:** 2022-11-09

**Authors:** Ahmed M. Basudan

**Affiliations:** Chair of Medical and Molecular Genetics Research, Department of Clinical Laboratory Sciences, College of Applied Medical Sciences, King Saud University, Riyadh 12372, Saudi Arabia; ahmbasudan@ksu.edu.sa

**Keywords:** breast cancer, incidence, patterns, Saudi Arabia, cancer registry, cancer trends

## Abstract

*Background and Objectives*: Breast cancer is considered the most commonly diagnosed type of cancer among women globally and in Saudi Arabia. This study aimed to assess breast cancer incidence patterns and trends among the Saudi female population. *Materials and Methods*: Breast cancer incidence parameters were obtained from the Saudi Cancer Registry (SCR). The data were retrospectively analyzed for the period from 2001 to 2017 to investigate changes in incidence rates. Temporal trends were also analyzed through joinpoint regression analysis and were dissected by age groups and administrative regions. *Results*: During the specified period, breast cancer jumped by 55% to constitute 30.9% of all cancer cases among Saudi females. The median age at diagnosis increased to reach 51 years at the end of that period, with an overall increase of 6.3%. The overall Age-Standardized Incidence Rate (ASR) escalated by 151.7% from 11.8/100,000 to 29.7/100,000 population for that period. The Eastern region noticeably had the highest ASR and peaked at 52.2/100,000 population. The joinpoint analysis of the ASR showed increased trends, with an annual percent change (APC) of 5.13% (*p* < 0.05, [95% CI 4–6.3]). An age-specific analysis was also performed and showed that the age group 70–74 years had the highest trend (APC 10.2%, [95% CI 7.2–13.4], *p* < 0.05). Region-specific analysis revealed that the Jouf region had the highest trend among the regions (APC 8.8%, [95% CI 3.7–14.2], *p* < 0.05). *Conclusions*: Our analysis indicates increased breast cancer incidence in Saudi Arabia with an alarming pace. With the existing trend, it is expected that Saudi Arabia will continue to display an increase in breast cancer incidence. Long-term preventive measures and more effective screening strategies are warranted to alleviate the burden of the disease.

## 1. Introduction

Breast cancer can be characterized as the abnormal proliferation of malignant cells in mammary epithelial and stromal and tissues. Over the years, breast cancer research has evolved tremendously, giving new insights into the disease’s diagnosis, development, progression, metastasis, and treatment. Despite remarkable advances in our understanding of the disease in the last half-century, it is still a major public health burden worldwide and poses a significant challenge. Breast cancer is considered the most commonly diagnosed invasive type of cancer among women globally [1]. Among women, it accounted for 24.5% of all cancer cases and 15.5% of cancer deaths around the world in 2020 [2]. In the United States, breast cancer is the leading cause of cancer death in women aged 20 to 59 years and the second-leading cause after lung cancer in women aged 60 years or older [3]. Unfortunately, patients with metastatic breast cancer (MBC) generally have a poor 5-year survival (5YS) rate of about 25%, which is still incomparable to the 5YS of localized disease (>95%) [4,5,6,7]. In Saudi Arabia, breast cancer was the most commonly diagnosed type of cancer and the second-leading cause of death after leukemia in 2018 [8]. Although the breast cancer incidence is much lower than it is many western countries, there is increasing evidence that incidence has been rising rapidly in Saudi Arabia [9]. Despite the fact that breast cancer constitutes a major public health burden in Saudi Arabia, the resources for epidemiological descriptions are limited compared to many western countries. This article provides a comprehensive assessment of breast cancer incidence parameters for the period from 2001 to 2017. Such information should be valuable for policy makers in implementing early detection and screening approaches to facilitate reduction in cancer mortality.

## 2. Materials and Methods

### 2.1. Data Source

Data are publicly available from the population-based Saudi Cancer Registry (SCR) under the supervision of the Saudi Health Council (SHC) and accessible through the National Health Information Center (NHIC) at https://nhic.gov.sa (accessed on 15 February 2022). The SCR oversees data collection through five regional offices which report to a main office to ensure full coverage of all health facilities around the kingdom. Records for 17 years (January 2001 to December 2017) were collected and examined retrospectively. Data for the years after 2017 were not publicly reported at the time of writing this manuscript. Relevant data including descriptive variables and other statistical parameters of Saudi female patients diagnosed with breast cancer for all age groups in all administrative regions were analyzed and reported. Cases with unknown nationalities and/or unknown International Classification of Diseases (ICD) codes, as identified by the World Health Organization (WHO), were excluded from the analyses. Cohort characteristics are illustrated in Table 1.

### 2.2. Administrative Regions of Saudi Arabia

**Figure 1:**
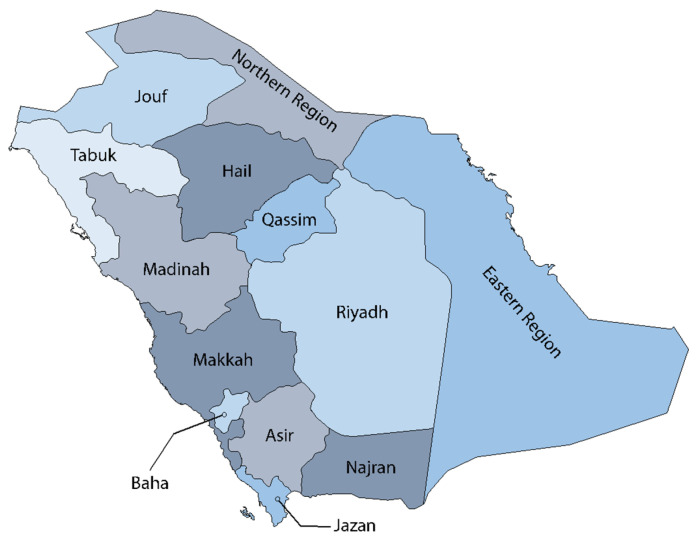
Administrative regions of Saudi Arabia. All of the 13 administrative regions are labeled in their location within the map (map modified from Vemaps.com).

Saudi Arabia has a land area of around 2.15 million km^2^ [10] and is divided into 13 administrative regions which include the Eastern Region, Riyadh, Makkah, Madinah, Najran, Tabuk, Northern Borders, Hail, Jouf, Asir, Qassim, Jazan, and Baha (Figure 1).

### 2.3. Statistical Parameters

All incidence parameters were stated as reported by the Saudi Cancer Registry (SCR). The Age-Specific Incidence Rate (AIR) was calculated as:

$$AIR=\frac{No.\;of\;cancer\;cases\;occurring\;during\;a\;specific\;period\;in\;a\;population\;of\;a\;specific\;age\;group}{No.of\;midyear\;population\;of\;that\;age\;group}$$

The Age-Standardized Incidence Rate (ASR) is a summary measure of a rate that a population would have if it had a standard (reference) age structure (the World Standardized Incidence Rate was used in this case). The ASR formula used was:

$$ASR=\frac{Total\;no.\;of\;expected\;cases}{Total\;standard\;poulation\;size}$$

The Crude Incidence Rate (CIR) was calculated as the total number of cases registered in a given time period as a proportion of the total population. CIR was calculated as:

$$CIR=\frac{Total\;no.\;of\;cases\;diagnosed\;in\;a\;specific\;year}{Total\;midyear\;population}$$

The incidence rates were expressed per 100,000 population.

The temporal trends estimations for age-standardized rates were predicted using the Joinpoint regression software ((version 4.9.1.0, https://surveillance.cancer.gov/joinpoint/ (accessed on 8 September 2022)) [11], which evaluates changing linear trends over successive segments of time. To reduce the possibility of the high variability in reporting changes in trends over time, the models were restricted to either 0 or 1 joinpoint. Trends were expressed as the annual percentage change (APC) at a significance level of alpha = 0.05 and a 95% confidence interval (CI). The method has been widely used to evaluate changes in incidence trends [12,13,14,15,16].

## 3. Results

### 3.1. Number of All Cancer Cases in the Saudi Population

**Figure 2:**
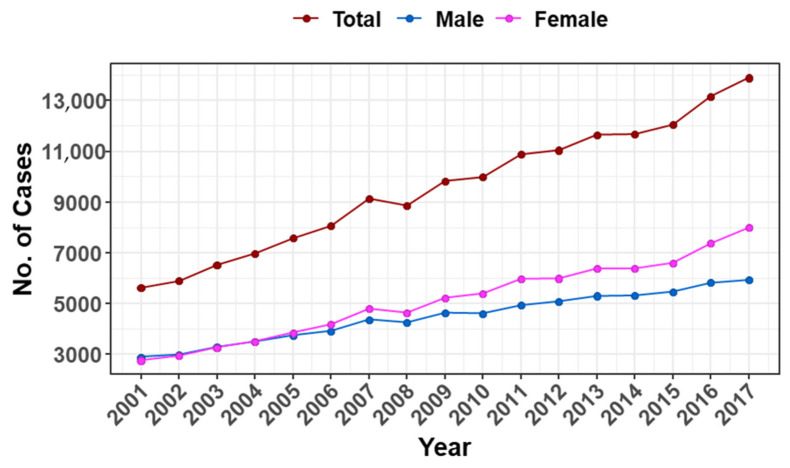
Distribution of cancer cases among the Saudi population by year. Shown are the number of all cancer cases (*y*-axis) for both genders (brown), males (blue), and females (pink) from 2001 to 2017, as indicated in the *x*-axis.

The total number of all cancer cases for both genders among Saudis in 2001 was 5616 and increased by 147.4% to reach 13,893 cases in 2017. For females only, 2741 cases were reported in 2001 to reach 7975 cases in 2017 (191% increase), whereas 2875 and 5918 cases were reported for males only in both years (105.8% increase), respectively (Figure 2).

### 3.2. Breast Cancer Cases and Age at Diagnosis in the Saudi Female Population

**Figure 3:**
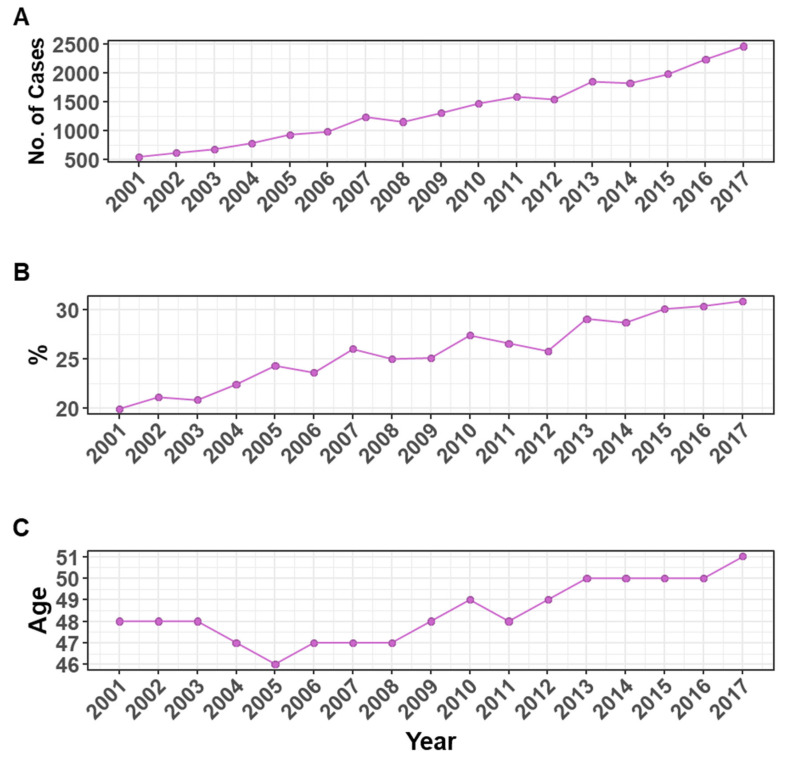
Breast cancer cases and age at diagnosis in the Saudi female population. (**A**) The number (*y*-axis) of breast cancer cases among the Saudi female population. (**B**) Percentage (*y*-axis) of breast cancer cases of all Saudi female cancers. (**C**) Median age at diagnosis indicated in the *y*-axis (for the years 2001–2003, only the mean was reported). The *x*-axis represents the years for the period of 2001–2017.

The number of breast cancer cases among Saudi females has increased by 351.9% from 545 cases in 2001 to 2463 in 2017 (Figure 3A and Table 2). During that period, breast cancer jumped by 55% from 19.9% to constitute 30.9% of all cancer cases among Saudi females (Figure 3B and Table 2). The median age at diagnosis was 48 years in 2001, dropped to 46 in 2015, and then kept increasing to reach 51 in 2017, with an overall increase of 6.3%, as indicated in Figure 3C and Table 2.

### 3.3. Incidence Rates for Breast Cancer among the Saudi Female Population

**Figure 4:**
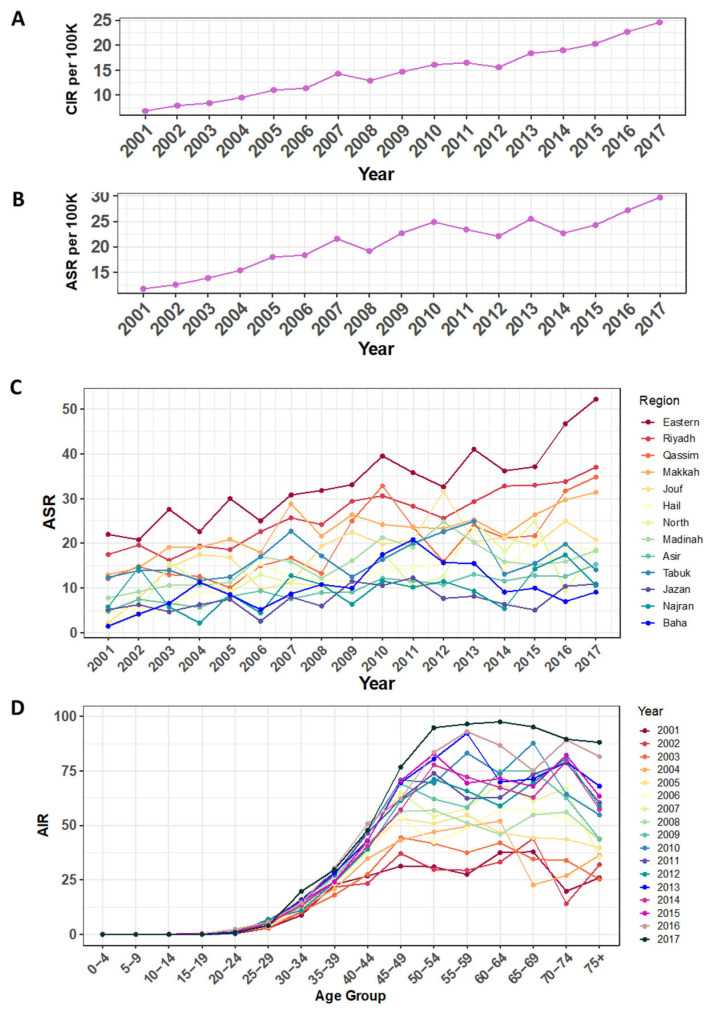
Incidence rates for breast cancer among the Saudi female population. (**A**) Breast cancer Crude Incidence Rate (CIR). (**B**) Overall Age-Standardized Incidence Rate (ASR). (**C**) Age-Standardized Incidence Rate (ASR) stratified by administrative regions in Saudi Arabia. The years for the period of 2001–2017 are indicated in the x-axes. (**D**) Age-Specific Incidence Rate (AIR) shown for each age group (*x*-axis). All rates are per 100,000 population, as indicated in the y-axes.

The breast cancer Crude Incidence Rate (CIR) had steadily increased from 6.8/100,000 to 24.6/100,000 for the period 2001–2017 (261.8% increase), as indicated in Figure 4A and Table 2. Similarly, the overall Age-Standardized Incidence Rate (ASR) had escalated by 151.7% from 11.8/100,000 to 29.7/100,000 for the same period (Figure 4B and Table 2). The ASR stratified by the Saudi administrative regions revealed multiple findings. Although some regions showed fluctuations for the period of 2001–2017, the Eastern region was clearly with the highest ASR and peaked in 2017 at 52.2/100,000. Conversely, regions such as Baha, Najran, and Jazan showed lower ASRs of 9.1, 10.6, and 10.9 per 100,000 for the same year, respectively (Figure 4C). The Age-Specific Incidence Rate (AIR) swung throughout the years for that period. However, the AIR has always reached its peak between the age of 50 and 74 years, except for year 2003, where the peak was in the 45–49 group (Figure 4D).

### 3.4. Distribution of Breast Cancer Histological Subtypes

The breast cancer cases were histopathologically subclassified into Invasive Ductal Carcinoma (IDC), Invasive Lobular Carcinoma (ILC), Mixed, or Other. For the period of 2001–2017, IDC constantly remained the dominant subtype (72.4–80.5%). The distributions for the other subtypes are indicated in supplementary Figure S1. The mean percentage for the IDC, ILC, and mixed subtypes were 77.8%, 5.8%, and 2.6%, respectively.

### 3.5. Trends in Breast Cancer Incidence

**Figure 5:**
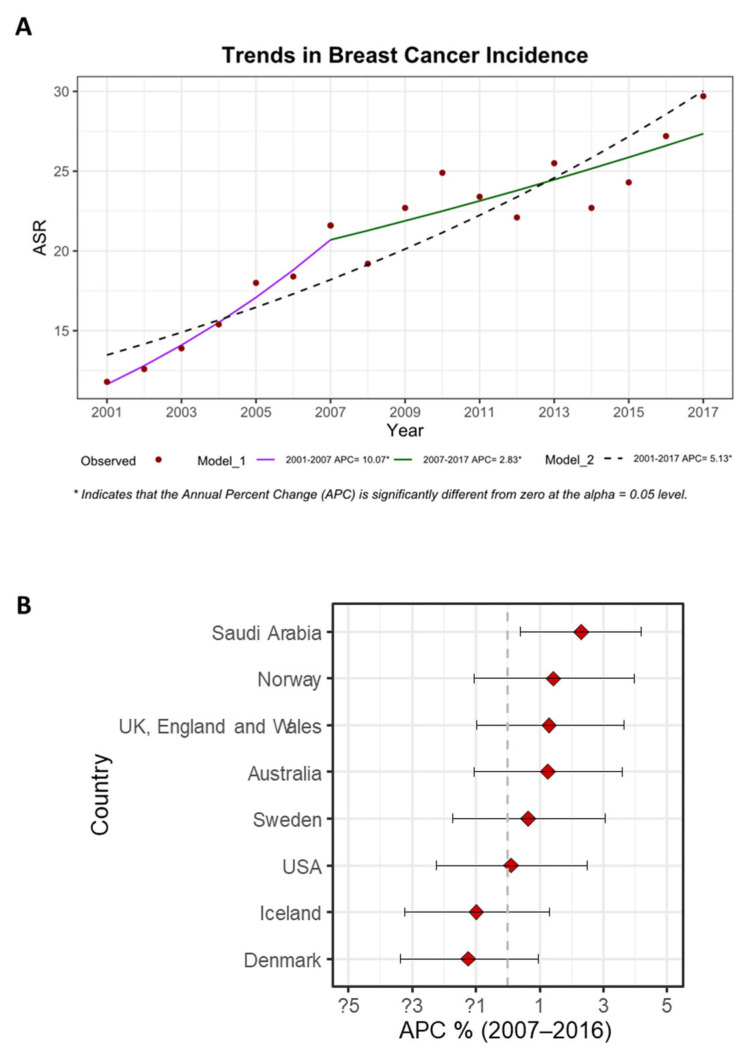
Trends in breast cancer incidence. (**A**) Jointpoint regression analysis of breast cancer Overall Age-Standardized Incidence Rate (ASR) in the Saudi female population for the period of 2001–2017. The estimated Annual Percent Change (APC) for two modules (Module_1 and Module_2) is displayed at the bottom of the figure, (* indicates *p* < 0.05). (**B**) Estimated Annual Percent Change (APC) of Overall Age-Standardized Incidence Rate (ASR) for selected countries in comparison to Saudi Arabia (2007–2016). Data for other countries were acquired from the International Agency for Research on Cancer (IARC) [17].

For the period of 2001–2017, a joinpoint analysis of the age-standardized incidence rates (ASR) of breast cancer showed increased trends, with an annual percent change (APC) of 5.13% (*p* < 0.05, [CI 4–6.3], Figure 5A). In another module, the trend was more pronounced for the period of 2001–2007 compared to the period of 2007–2017 (APC 10.07% [CI 6.3–14] vs. 2.83% [CI 1.2–4.5], Figure 5A). An age-specific analysis was also performed and showed that the age group of 70–74 years had the highest trend (Table 3: APC 10.2%, [CI 7.2–13.4], *p* < 0.05). Region-specific analysis revealed that the Jouf region had the highest trend among the regions (Table 4: APC 8.8%, [CI 3.7–14.2], *p* < 0.05).

Estimated APCs in ASR for selected countries were available for the period of 2007–2016 through the International Agency for Research on Cancer (IARC) [17]. For Saudi Arabia, our joinpoint analysis for the same period revealed an APC of 2.3% (*p* < 0.05, [CI 0.4–4.2]), which was the highest compared to the rest of the countries (Figure 5B).

## 4. Discussion

This study describes a comprehensive assessment of female breast cancer incidence by age and geographical distribution for the period of 2001–2017 in Saudi Arabia. Overall, the burden of breast cancer incidence is rapidly growing, where the number of breast cancer cases among Saudi females increased by 351.9%. During the same period, breast cancer jumped by 55% from 19.9% to constitute 30.9% of all cancer cases among Saudi females. The breast cancer Crude Incidence Rate (CIR) underwent a 261.8% increase to reach 24.6 per 100,000 in 2017. Similarly, the overall Age-Standardized Incidence Rate (ASR) has escalated by 151.7% and peaked at 29.7/100,1000 in that year. A deeper analysis of the ASR showed some variation between the different geographical regions. For example, the Eastern and Riyadh regions reported the highest incidence, in contrast to regions such as Baha and Jazan. This variation can be explained by the fact that the Riyadh and Eastern regions represent the largest two regions in Saudi Arabia, and they hold more than 40% of the population. In addition, this increase can be attributed—at least partly—to higher accessibility to better health care facilities, where a higher detection rate for cases is expected [18].

It has previously been reported that more than 55–75% of breast cancer cases among Arab women are diagnosed before the age of 50 years (in contrast to only 30% in western countries) [19,20]. Interestingly, the median age at diagnosis in our analysis showed an interesting increase of 6.3% and reached 51 years in 2017. This finding seems to be in line with the current transition to and adaptation of a more westernized lifestyle in Saudi Arabia. Although the ASR in Saudi Arabia might seem low compared to many western countries, our analysis of the annual percent change (APC) of ASR for a ten-year period (2007–2016) in Saudi Arabia showed an alarming rate in comparison to selected countries (Saudi Arabia was at the top of the list with 2.3% APC). The marked increase in breast cancer incidence in Saudi Arabia is worth exploring. It is possible that changes in factors such as reproductive capacity, socioeconomic status, life expectancy, smoking, screening, and treatment options can lead to an accelerated increase in breast cancer incidence. It has been previously estimated that women who have their first full-term pregnancy before the age of 20 have a significantly lower risk of developing breast cancer compared to those after the age of 30, and the risk is more pronounced among estrogen-receptor-positive cases [21,22]. The last couple decades have shown marked changes in the roles of Saudi women, which has affected reproductive behavior, particularly the age at first childbirth. In that period, the mean age first pregnancy increased tremendously from 18 to 28 years [23,24]. In addition, fertility has declined by more than 50% in Saudi Arabi from 1990 to 2014 [19]. There is a significant amount of evidence that multiple full-term pregnancies can reduce the risk of developing breast cancer [25,26].

Sedentary lifestyle (accompanied by obesity, diabetes, and decreased physical activity) among women in Saudi Arabia and the Gulf Cooperation Council (GCC) countries is high and can lead to increased breast cancer risk. The reduced physical activity is probably due to the limited access to suitable exercise facilities, cultural norms in some regions, and the extremely hot weather that extends to many months during the year [27,28,29]. In addition, the abundance of housemaids and drivers to take care of many of the household duties can be contributing factors. There is clear evidence that the relative risk (RR) of developing breast cancer is more than twice as high for obese women compared to normal-weight women, especially in postmenopausal females [30,31,32].

With the adoption of a westernized lifestyle, smoking status and exposure to stress are expected to increase among Saud females. In fact, the estimated incidence for smoking in both genders has increased by 12.5% (the highest among GCC countries) from 1990 to 2012 and is expected to increase further [9]. In addition, smoking shisha—which is more preferable to females than cigarettes—is far worse because of the much higher amounts of nicotine [33]. It has been evidenced that women who have been smoking for more than ten years have about a 10% increased risk of developing breast cancer compared to non-smoking women [34,35,36].

As a result of the better control of communicable and infectious diseases, Saudi Arabia has shown a longer life expectancy. The total life expectancy consistently increased by 7.4% from 1990 to 2014 [19]. Most cancers (including breast cancer) are considered age-related disease, and the incidence increases with age and longevity [37,38].

To our knowledge, this study represents the longest evaluation of breast cancer incidence patterns in the Saudi female population. Nevertheless, there is an urgent need for information about mortality trends and survival rates, which are not reported here. Given that mortality has declined in many westernized countries [39], it will be interesting to explore if the same behavior is recognized in Saudi Arabia. Similarly, information about the stage at the diagnosis is important, as it reflects the effect on prognosis. It has been shown that Arab women with breast cancer fail to seek medical attention at early phases, even if the disease is symptomatic. This, in turn, was reflected in the high number of aggressive cases seen in healthcare centers. However, it is expected that, with more awareness and education programs, there will be an increase in the early detection of cases, which will increase the case incidence but lead to less aggressive behavior. It is well-known from multiple studies that breast cancer presentation in young women is more likely to be at an advanced stage and aggressive [40,41,42,43]. With the change seen in the mean age at diagnosis (now >50 years), it is pivotal to perceive how that correlates with the aggressiveness and stage of the disease. Finally, it has been shown that BRCA mutant breast cancer is more likely to be triple negative (TNBC) and more aggressive than sporadic breast cancer [44,45]. Thus, it is essential in monitoring how BRCA mutations frequency changed over the years given that there might have been changes in the consanguinity rates. This change can affect the molecular subtypes distribution and, eventually, the treatment options and mortality.

## 5. Conclusions

Our analysis indicates increased breast cancer incidence in Saudi Arabia, concomitant with changes in routine habits and the adoption of a westernized lifestyle. With current data trends, it is expected that Saudi Arabia might display an increase in breast cancer incidence until it reaches a stable ASR—similar to or even more than that seen in many western countries. Therefore, long-term preventive measures and strategies should be applied to reduce breast cancer incidence and mortality. These include (but are not limited to) combating tobacco use, increasing awareness of body weight and physical activity, and implementing effective screening programs.

## Figures and Tables

**Table 1:** Characteristics of the samples included in the study.

Nationality	Gender	Subtypes	Age
Saudi only	Female only	Invasive Ductal Carcinoma (IDC), Invasive Lobular Carcinoma (ILC), Mixed, or Other	All age groups, as follows: 0–4, 5–9, 10–14, 15–19, 20–24, 25–29, 30–34, 35–39, 40–44, 45–49, 50–54, 55–59, 60–64, 65–69, 70–74, and >75

**Table 2:** Summary of Breast Cancer Incidence Parameters Among the Saudi Female Population.

Parameter	2001	2017	% Increase (2001–2017)
Total number of all cancer cases	2741.0	7975.0	191.0
Total number of breast cancer cases	545.0	2463.0	351.9
% of breast cancer cases among all cancer cases	19.9	30.9	55.3
Breast cancer median age at diagnosis	48.0	51.0	6.3
CIR	6.8	24.6	261.8
ASR	11.8	29.7	151.7

Abbreviations: CIR = Breast cancer overall crude incidence rate; ASR = Breast cancer overall age-standardized incidence rate.

**Table 3:** Trends in Breast Cancer Incidence by Age Groups (2001–2017).

Age Group	APC	95% CI
45–49	4.1 *	[2.6–5.7]
50–54	6.8 *	[5.7–8]
55–59	6.9 *	[5.1–8.7]
60–64	5.2 *	[3.6–6.9]
65–69	6.0 *	[3.4–8.6]
70–74	10.2 *	[7.2–13.4]
75+	7.2 *	[6–8.5]

Abbreviations: APC = Annual Percentage Change; CI = Confidence Interval. * Indicates that the Annual Percent Change (APC) is significantly different from zero at the alpha = 0.05 level.

**Table 4:** Trends in Breast Cancer Incidence by Administrative Regions (2001–2017).

Region	APC	95% CI
Asir	6.0 *	[4.6–7.5]
Baha	6.9 *	[1.1–13.1]
East	4.8 *	[3.7–6]
Hail	8.7 *	[6.2–11.3]
Jazan	3.8	[−0.1–7.8]
Jouf	8.8 *	[3.7–14.2]
Madinah	4.8 *	[2.5–7.3]
Makkah	4.0 *	[2.4–5.6]
Najran	4.7	[−0.5–10.2]
North	8.6 *	[3–14.6]
Qassim	6.2 *	[3.6–8.9]
Riyadh	4.8 *	[3.8–5.9]
Tabuk	2	[−0.4–4.4]

Abbreviations: APC = Annual Percentage Change; CI = Confidence Interval. * Indicates that the Annual Percent Change (APC) is significantly different from zero at the alpha = 0.05 level.

## Data Availability

Publicly available datasets were analyzed in this study. These data can be found here: https://nhic.gov.sa (accessed on 15 February 2022).

## Supplementary Materials

The following supporting information can be downloaded at: https://www.mdpi.com/article/10.3390/medicina58111617/s1, Figure S1: Distribution of breast cancer histological subtypes. Percentage of each subtype is shown in the *y*-axis and color-coded (dark red = IDC, orange = ILC, yellow = Mixed, and grey = Other). The *x*-axis represents the years for the period of 2001–2017. IDC: Invasive Ductal Carcinoma; ILC: Invasive Lobular Carcinoma.
