# Supplementary material for: Breast Cancer Incidence Patterns in the Saudi Female Population: A 17-Year Retrospective Analysis
Source: Medicina (Kaunas). 2022 Nov 9;58(11):1617. doi: 10.3390/medicina58111617 (PMC9697748; doi:10.3390/medicina58111617)
Supplement: Supplementary file 1 [file medicina-58-01617-s001.zip › medicina-1980428-supplementary.pdf]

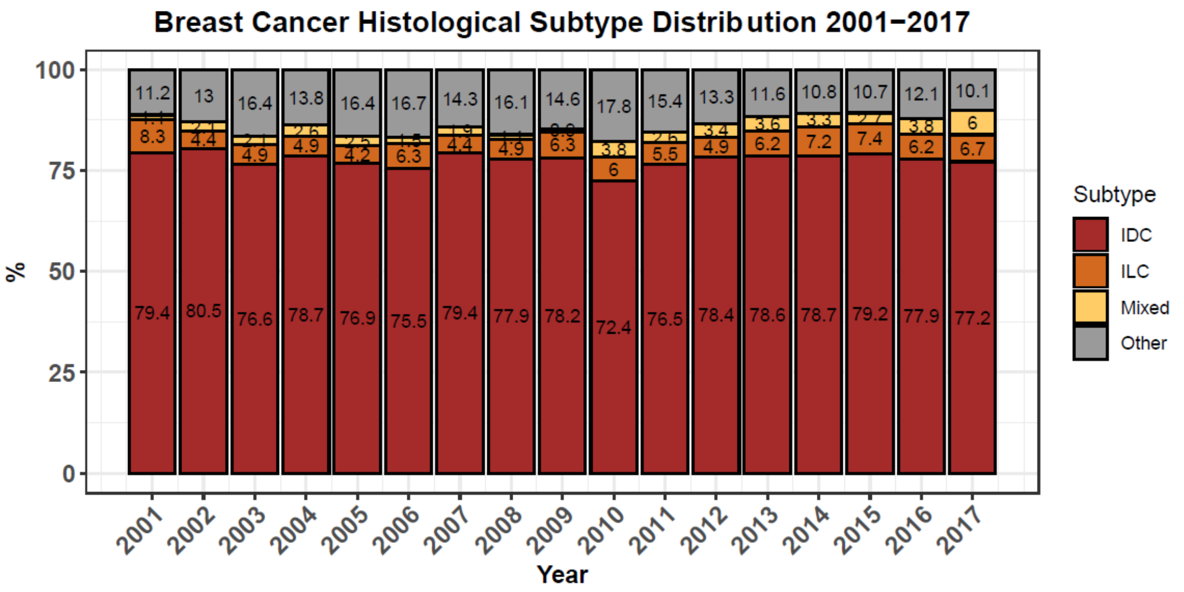

Figure S1. Distribution of breast cancer histological subtypes. Percentage of each subtype is shown in the *y*-axis and color-coded (dark red = IDC, orange = ILC, yellow = Mixed, and grey = Other). The *x*-axis represents the years for the period of 2001–2017. IDC: Invasive Ductal Carcinoma; ILC: Invasive Lobular Carcinoma.
